# Death Anxiety, Reliability, Validity, and Factorial Structure of the Farsi Form of the Arabic Scale of Death Anxiety in Iranian Old-Aged Persons

**DOI:** 10.1155/2016/2906857

**Published:** 2016-10-27

**Authors:** Mahboubeh Dadfar, David Lester, Fazel Bahrami

**Affiliations:** ^1^School of Behavioral Sciences and Mental Health, Tehran Institute of Psychiatry, Iran University of Medical Sciences, International Campus, Tehran, Iran; ^2^Department of Psychology, Stockton University, Galloway, NJ, USA; ^3^Department of Islamic Theology and Department of Counseling, University of Social Welfare and Rehabilitation Sciences, Tehran, Iran

## Abstract

The present study is aimed at examining the level of death anxiety and the sex-related differences among old-aged Iranian individuals sample to compare the old-aged persons with young college students and to explore the psychometric properties of the Arabic Scale of Death Anxiety (ASDA) factors in old-aged sample. A sample of 146 volunteer Iranian individuals took part in the study. The mean ages were 68.58 (SD = 7.10), men 68.81 (SD = 7.44) and women 68.28 (SD = 6.76), respectively. The mean score of the ASDA was 51.09 (SD = 20.19). Cronbach's alpha of the ASDA was found to be high (0.94); and Spearman-Brown coefficient was 0.92. Women had a significantly higher mean total score on the ASDA. Old-aged individuals had a significantly higher mean ASDA total score than younger college students (M age = 25.77). The factor analysis of the ASDA items yielded three factors accounting for 67.88% of the total variance labeled (F1) fear of dead people and tombs; (F2) fear of lethal disease and postmortem events; and (F3) death fear. These factors were highly replicable with previous factors extracted from a middle-aged Kuwaiti sample. On the basis of the present results, there are the following three general conclusions: death anxiety is not significantly correlated with age; the sex-related differences on death anxiety are striking in the Iranian samples; and the ASDA has a highly replicable factor structure among two Iranian and Arab countries.

## 1. Introduction

Death anxiety is a multifaceted construct. It was defined and described the different ways in which it is manifested [1–3]. Death anxiety has been conceptualized as fear of death of oneself; fear of death of others; fear of dying of self; and fear of the dying of others [4–7]. Many factors influence death anxiety such as age, sex, culture, religion, physical health, and mental health [2]. Aging is a stage in developmental psychology and associated with various medical problems, loss of loved ones, and deteriorating cognitive abilities. Old-aged individuals being nearer to the end of life may experience death anxiety or death fear [8, 9].

Cultural variations in conceptualizations of death can determine death anxiety in different ages [10–12]. Many researches showed that death anxiety exists among different cultures including Iranian culture [8, 13–15]. Missler et al. revealed that Dutch elderly people who were in an assisted living facility had higher levels of fear for others and of the dying process than of fear of the unknown. The fear for important others correlated with poor somatic health; and fear of the dying process correlated with low self-esteem, small goal in life, and poor mental well-being [16]. For most of old-aged individuals who encounter death of their spouses, death anxiety increases in them. For example, Momtaz et al., on a national cross-sectional survey in Malaysian middle-aged and older couples, reported that women (52%) had significantly higher spousal death anxiety compared to men (45%). Caregiving issues for older men and financial security for older women could predict increasing spousal death anxiety [17].

Death anxiety impacts behavior and life and death decisions [18, 19], and one study found that depression and severity of illness positive correlated with death anxiety [20]. One study showed that death anxiety could be a determining factor of health promoting behaviors both in young and older adults [21]. Abdel-Khalek and Lester found that somatic symptoms were associated with death anxiety, on the Arabic Scale of Death Anxiety (ASDA) and the Collett-Lester Fear of Death Scale (CLFDS); the Kuwaiti young adults with good somatic health were anxious about death compared with American young adults [22].

The ASDA has been used in various populations, for example, health professionals [23], drug dependents [24], psychiatric patients, healthy individuals [25], college students [3], and middle-aged individuals [26], to investigate the factors related to death anxiety.

In spite of the fact that death anxiety is one of the issues related to health, well-being, and welfare of elderly persons in the societies, the little studies have focused on it among the elder population. In the present study it is hypothesized that (a) Iranian old-aged women would have higher death anxiety, (b) death anxiety levels would differ between Iranian young adults and older adults, and (c) and (d) the Farsi form of the Arabic Scale of Death Anxiety (ASDA) has good psychometric properties of factors among Iranian old-aged individuals.

## 2. Methods

### 2.1. Participants

The study was a cross-sectional, descriptive study. A sample of 146 volunteer Iranian old-aged individuals took part in the study. They were selected by convenient sampling. The elders had come to the neighborhood parks for leisure, recreation, and the activities done for enjoyment when they were not working. The study is limited to the old persons located in Tehran city. The range of age was 60–90 years, with mean ages 68.58 (SD = 7.10), male being 68.81 (SD = 7.44) and female being 68.28 (SD = 6.76), respectively. 80 (58%) were male and 58 (42%) female. The majority of them were married (*n* = 107; 75%) and had an education level of diploma (*n* = 56; 39%).

### 2.2. Measures

Demographic characteristics sheet of the sample includes age, gender, education status, and marital status. The Arabic Scale of Death Anxiety (ASDA) was developed by Abdel-Khalek, and it is one of the scales for measuring death anxiety [27]. The ASDA has been validated in the Arabic countries of Egypt, Kuwait, and Syria [26–28], the Western countries [29], and the Asian countries of Turkey [3]. It has 20 items, and each item is answered on a five-point intensity scale anchored by no (1) and very much (5) [28].

Abdel-Khalek and Al-Kandari reported that Cronbach's alpha of the ASDA was high (0.93), and the ASDA yielded three factors disease in Kuwaiti middle-aged individuals labeled (F1) fear of dead people and tombs; (F2) fear of postmortem events; and (F3) fear of lethal disease [26]. Sarıçiçek Aydoğan et al. found Cronbach's alpha for Turkish version of the ASDA was 0.86, for each item was from 0.85 (for items (11) and (12)) to 0.90 (for item (20)), the Guttman coefficient was 0.86, and the ASDA was highly associated with the DAS (*r* = 0.68). They obtained five factors in the sample of Turkish college students, accounting for 65.6% of the total variance and labeled (F1) fear provoked by visual stimuli related to death; (F2) fear of physical and psychological pain related to death; (F3) fear of other situations reminding of death; (F4) fear of postmortem events; and (F5) fear of the act of dying [3].

## 3. Results

The Pearson correlations between the total score of the ASDA and age was 0.012 (no significance); sex −.443 (*p* > .01); education −.155 (no significant); and marital status .302 (*p* > .01).

### 3.1. Reliability of the ASDA

For old-aged individuals' sample, the Cronbach alpha was 0.94, the split-half reliability was 0.86, the Spearman-Brown coefficient was 0.92, and the Guttman coefficient was 0.92. The intercorrelations between the items ranged from 0.15 to 0.79, and the item-total correlations ranged from 0.60 to 0.80 in the sample.

The mean score of the ASDA was 51.09 (SD = 20.19), for women 62.36 (SD = 17.00) and men 44.33 (SD = 18.93). Old-aged women had a significantly higher mean ASDA total score than men (*t* = 5.75, *p* < .001). The mean scores of the ASDA items were from 1.67 (SD = 1.10) for item (12) “I dread walking in graveyards” to 3.58 (1.49) for item (6) “I worry that death deprives me of someone dear to me.” Old-aged women had significantly higher mean scores in all of the ASDA items than men (Table 1).

The mean score of the ASDA for elders was 51.09 (SD = 20.19) and for college students 41.40 (SD = 12.73). Old-aged individuals had a significantly higher mean ASDA total score than younger college students (*t* = −5.86 and *p* < .001) (Table 2).

### 3.2. Factor Analysis of the ASDA

The criteria for the factor analysis were evaluated using the Kaiser-Meyer-Olkin Measure of Sampling Adequacy (KMO) and the Bartlett Test of Sphericity. The KMO was 0.919, indicating the adequacy of the sample of college students, and the Bartlett Test of Sphericity was 2.203*E*3 (df = 190 and *p* < .001) indicating that the factor analysis was justified in the elders sample. Three components with eigenvalues greater than one were retained in the sample of elders as reported in Table 3.

Factor 1 (8 items) explained 49.71% of the observed variance and was labeled “fear of lethal disease and death.” It included the following items: “I fear looking at the dead,” “I am afraid of looking at a corpse,” “witnessing the burial procedure terrifies me,” “I dread walking in graveyards,” “I am preoccupied with thinking about what will happen after death,” “I get upset by witnessing a funeral,” “the sight of a dying person frightens me,” and “talking about death upsets me.”

Factor 2 (8 items) explained 11.95% of the observed variance and was labeled “fear of lethal disease and postmortem events” and included the following items: “the possibility of having a surgical operation terrifies me,” “I am afraid of suffering heart attack,” “I worry that death deprives me of someone dear to me,” “I am apprehensive of unknown things after death,” “I fear getting a serious disease,” “I am preoccupied with thinking about what will happen after death,” “the pain accompanying death terrifies me,” and “I am afraid of getting cancer.”

Factor 3 (4 items) explained 6.20% of the observed variance and was labeled “death fear”. It included the following items: “I fear death whenever I because ill,” “I fear visiting graves,” “I am afraid of sleeping and not waking up again,” and “I fear death”.

## 4. Discussion and Conclusion

The first hypothesis was confirmed. The study showed that female Iranian elders had a significantly higher mean ASDA total score compare to men. The gender and age results in this Iranian sample are similar to that in some Western, Arabic, and Indian samples. For example Neimeyer and Fortner found that women had higher death anxiety than men [30]. Suhail and Akram showed that Pakistani Muslims women had higher death anxiety than men [31]. Abdel-Khalek using the ASDA declared that Egyptian women in clinical and nonclinical groups had higher mean scores than men [25]. Another study showed that there were strong sex differences in the DAS scores of men and women [11]. Abdel-Khalek and Al-Kandari found Kuwaiti middle-aged women had a significantly higher mean ASDA total score also on 17 out of 20 items of the scale [26]. On the Death Anxiety Survey Schedule from an Indian perspective, Madnawat and Kachhawa indicated that women were significantly more anxious about the word of death [32]. Abdel-Khalek et al. found that, on the ASDA, Egyptian, Kuwaiti, Lebanese, Syrian, Spanish, and American women (no English women) had significant higher mean scores than men [29]. Missler et al. reported that women compared to men had more fear of the death of loved ones and of the consequences of their own death on these loved ones [16]. Singh reported that women as compared with men had more death anxiety [33]. Thabet et al. found that Palestinian women had more death anxiety than men [34]. Our results were not similar to findings of Fortner and Neimeyer that reported gender did not predict death anxiety in elderly people but death anxiety was higher in young female adults [35]; Wu et al. showed that gender does not affect death anxiety in Chinese elderly people [36]; Chuin and Choo found that Malaysian women had lower death anxiety [37]; and Mimrot indicated that gender had no impact on death anxiety; and there was no significant difference between Indian old age men and women in death anxiety scores [38]. Rasmussen and Johnson found female undergraduate college students had higher levels of death anxiety than males [39]. On the Death Anxiety Scale (DAS), Death Depression Scale (DDS), and Death Obsession Scale (DOS), Bahrami et al. reported that Iranian Muslim female older adults had higher death distress than men but no significant difference [8]. Overall, it seems that Iranian elder women were more expressive of their death anxiety, and elder men were more in denial of their death anxiety. Schumaker et al. indicated that causes of higher death anxiety in women include encouraging to pursue success and attain accomplishments for men in many cultures, more readily admitting troubling feelings in women, and different connotations and implications for men and women [40]. Chuin and Choo indicated that men like women have death anxiety but suppress it or deny it [37]; also process of socialization [25], emotional evaluation of death by women, and cognitive evaluation of death by men can justify lower death anxiety in men [41].

The second hypothesis was affirmed. Our findings showed that old-aged individuals had a significantly higher mean ASDA total score than younger college students. Suhail and Akram showed that Pakistani Muslim elders had higher death anxiety [31]. Mimrot found that Indian old age people had high death anxiety. Some studies reported findings inconsistent with our results [38]. Keller et al. reported that middle age and late-middle age individuals were significantly less anxious in regard to evaluation of death in general than older and younger persons. Old age individuals had the least anxiety toward death anxiety related to self [42]. Studies of Feifel and Nagy; Maiden and Walker; Walker and Maiden, cited in Chuin and Choo, declared that age was not related to death fear and age did not impact death anxiety as elders were no more likely to fear death compared to younger persons [37]. Singh reported that younger persons as compared with older persons had more death anxiety [33]. Goebel and Boeck, cited in Mimrot, reported low death anxiety in late adulthood; and age was not a significant factor in determining the degree of death anxiety in late adulthood [38]. Rasmussen and Brems indicated that psychosocial maturity was a stronger predictor of death anxiety than age; and both age and psychosocial maturity were significantly inversely related to death anxiety [43]. Abdel-Khalek and Al-Kandari found that Kuwaiti middle-aged individuals had a significantly lower mean ASDA total score than younger college students [26]. Chuin and Choo revealed that death anxiety levels did not differ between Malaysian young adults and older adults [37].

There was a linear increase in death anxiety along with the advance in age [44]. Goebel and Boeck, cited in Mimrot, found no relationship between death anxiety and age [38]. One study found that individuals with physical and mental health problems are more sensitive to death anxiety [41]. Another study showed that Indian old age people living in the family had high death anxiety. There was a difference between Indian old age people living in family and old age institutionalized people in death anxiety; and old age people living in the family had high death anxiety [38]. One study indicated that elders living in the more dangerous situations had higher death anxiety than those living in less dangerous situations [33]. Overall, death anxiety levels change according to used tools for death anxiety, place of living, and lifestyle of old-aged persons, and are also related to experiences that elders had during their lives.

Mimrot concluded that ego integrity or an accomplishment and fulfillment of personal sense, founded in religion or some other related measures, is probably the greatest factor in death anxiety [38]. Our finding may reflect cultural differences in death anxiety. Also our study did not support Erikson's theory because the Iranian old-aged sample could not be viewed in the ego integrity stage of Erikson's theory and they experienced high death anxiety. It is probable that an old person can equilibrate for psychosocial maturity in Rasmussen-Brens's theory with ego integrity Erikson's theory, so we can consider supporting Erikson's studies in Iranian old-aged persons.

The third hypothesis of the present study was to explore the reliability, validity, and descriptive statistics of the Farsi version of Abdel-Khalek's ASDA in the Iranian samples of elders. The hypothesis was proved. The results indicate that the ASDA has high internal consistency, split-half, and Spearman-Brown reliabilities (ranging from 0.86 to 0.94) in the sample. The principal components analysis identified three components of the ASDA in Iranian elders as follows: “fear of dead people and tombs,” “fear of lethal disease and postmortem events,” and “death fear.” These factors were highly replicable with previous high internal consistency and factors extracted from a Kuwaiti college student sample and a Kuwaiti middle-aged sample [26]. Abdel-Khalek obtained four components in an Egyptian sample, labeled “fear of dead people and tombs,” “fear of postmortem events,” “fear of lethal disease,” and “death preoccupation.” The first two factors were almost completely identical in three Arab countries [28]. Sarıçiçek Aydoğan et al. obtained five factors in a Turkish college students sample, labeled “fear provoked by visual stimuli related to death,” “fear of physical and psychological pain related to death,” “fear of other situations reminding death,” “fear of postmortem events,” and “fear from the act of dying” [3]. There were some similarities in specific factors between the previous results and the present findings.

In the present study, mean score of the ASDA was 51.09 (SD = 20.19). In study of Chuin and Choo mean score of the DAS was 7.35 (SD = 3.49) in middle adulthood and elders [37]; and, in study of Bahrami et al. (2014), mean score of the DAS was 7.48 (SD = 3.32) in Iranian elders [8]. The ASDA items receiving the highest mean score in the present sample of old ages were as follows: item (5) “I am afraid of suffering heart attack” (3.07), item (6) “I worry that death deprives me of someone dear to me” (3.58), item (15) “The pain accompanying death terrifies me” (3.19), item (10) “I fear getting a serious disease” (3.32), and item (19) “I am afraid of getting cancer” (3.47).

Religious attitudes are one of the correlates of death anxiety in aging. One study found that religiousness buffered against the death and dying anxiety in American late adulthoods [45]. Several studies investigated the relationship between religiosity and death anxiety [46, 47]. Rasmussen and Johnson showed that spirituality had a significant negative relationship with death anxiety [39]. Chuin and Choo showed that religious individuals did not have lower death anxiety [37]. Dadfar et al. found that there was no significant association between religious spiritual well-being and death anxiety total scores among Iranian Muslim elders [9]. In view of Pyne, relationship between religiosity and death fear is consistent with rational choice theory [48].

Our present findings showed that death anxiety has no significant correlation with age; the sex-related differences on death anxiety are striking in the Iranian samples; and the ASDA has a highly replicable factor structure. These results are similar to findings of Abdel-Khalek and Al-Kandari of the volunteer Kuwaiti middle-aged individuals [26]. Therefore, we conclude that the ASDA is useful for assessing death anxiety in Iranian elders in clinical and nonclinical samples.

The present results must be viewed within the limitations imposed by the data. Despite the good psychometric characteristics of the ASDA, the recruitment of the sample was not random but rather one of convenience. The sample was related to the population who live in Tehran, the capital of Iran and Tehran Province, in the north of the country, with a population of around 9 million in the city and 16 million in the wider metropolitan area. It is known that population who live in Tehran may differ from other regions in the country of Iran. Religious spiritual attitudes can predict death anxiety in old-aged individuals, but they were not assessed in our study. However, the ASDA may be useful in research on death anxiety among Iranian old-aged and middle-aged persons, and it is hoped that this study will stimulate further cross-cultural research on the ASDA in the elders.

Future research should be differentiated between the concept of death and the process of dying. Constructing validity of the ASDA with the other scales, using Multidimensional Fear of Death Scale (MFODS), Death Attitude Profile-Revised (DAPR), longitudinal and qualitative studies, comparing old age people living in institutions with old people living in the family, improving the quality of life and health care services for elderly, and investigating death anxiety and factorial structure of the ASDA on the Iranian middle-aged population, physical patients, addicted patients, and the assessing of components and correlates of demographic, psychological, social, religious spiritual, and subcultural of death anxiety in future studies are recommended.

## Figures and Tables

**Table 1:** Mean and standard deviation of the Arabic Death Anxiety Scale items in Iranian elders.

(ASDA) items	M (SD)	M (SD)	M (SD)	*t*	*p*
	Total	Women	Men		
(1) I fear death whenever I because ill.	2.41 (1.45)	2.89 (1.44)	2.12 (1.39)	3.16	.002
(2) I fear looking at the dead.	1.95 (1.26)	2.50 (1.48)	1.63 (.97)	4.11	.000
(3) I fear visiting graves.	2.01 (1.39)	2.65 (1.58)	1.62 (1.09)	4.51	.000
(4) The possibility of having a surgical operation terrifies me.	2.58 (1.49)	3.29 (1.33)	2.11 (1.41)	4.95	.000
(5) I am afraid of suffering heart attack.	3.07 (1.45)	3.77 (1.10)	2.62 (1.47)	4.99	.000
(6) I worry that death deprives me of someone dear to me.	3.58 (1.49)	4.12 (1.07)	3.30 (1.60)	3.38	.001
(7) I am apprehensive of unknown things after death.	2.79 (1.57)	3.43 (1.35)	2.40 (1.57)	4.02	.000
(8) I am afraid of looking at a corpse.	1.90 (1.29)	2.34 (1.52)	1.65 (1.05)	3.15	.002
(9) I fear the torture of the grave.	2.99 (1.55)	3.75 (1.32)	2.57 (1.51)	4.76	.000
(10) I fear getting a serious disease.	3.32 (1.45)	3.98 (1.08)	2.93 (1.50)	4.51	.000
(11) Witnessing the burial procedure terrifies me.	1.97 (1.35)	2.37 (1.53)	1.67 (1.12)	3.11	.002
(12) I dread walking in graveyards.	1.67 (1.10)	2.01 (1.31)	1.46 (.88)	2.95	.004
(13) I am preoccupied with thinking about what will happen after death.	2.57 (1.51)	3.22 (1.45)	2.20 (1.42)	4.13	.000
(14) I am afraid of sleeping and not waking up again.	2.34 (1.59)	2.93 (1.75)	1.93 (1.31)	3.80	.000
(15) The pain accompanying death terrifies me.	3.19 (1.43)	3.75 (1.04)	2.85 (1.55)	3.84	.000
(16) I get upset by witnessing a funeral.	2.08 (1.33)	2.48 (1.42)	1.87 (1.23)	2.66	.009
(17) The sight of a dying person frightens me.	2.45 (1.40)	3.13 (1.35)	2.10 (1.27)	4.58	.000
(18) Talking about death upsets me.	2.14 (1.36)	2.63 (1.43)	1.87 (1.26)	3.30	.001
(19) I am afraid of getting cancer.	3.47 (1.54)	4.03 (1.12)	3.16 (1.68)	3.42	.001
(20) I fear death.	2.51 (1.60)	3.00 (1.62)	2.21 (1.52)	2.91	.004
*Total score of the ASDA*	51.09 (20.19)	62.36 (17.00)	44.33 (18.93)	5.75	.000

**Table 2:** Mean and standard deviation of the Arabic Death Anxiety Scale in Iranian elders (*N* = 146) and college students (*N* = 252).

Arabic Scale of Death Anxiety (ASDA)	Elders	College students	*t*	*p*
	M (SD)	M (SD)		
	51.09 (20.19)	41.40 (12.73)	−5.86	.000

**Table 3:** Factor loadings of the Farsi version of the Arabic Scale of Death Anxiety (ASDA) in Iranian elders (*N* = 146).

(ASDA) items	Component		
	1	2	3
(1) I fear death whenever I because ill.			**.707**
(2) I fear looking at the dead.	**.754**		
(3) I fear visiting graves.			**.777**
(4) The possibility of having a surgical operation terrifies me.		**.610**	
(5) I am afraid of suffering heart attack.		**.782**	
(6) I worry that death deprives me of someone dear to me.		**.631**	
(7) I am apprehensive of unknown things after death.		**.506**	
(8) I am afraid of looking at a corpse.	**.788**		
(9) I fear the torture of the grave.			
(10) I fear getting a serious disease.		**.862**	
(11) Witnessing the burial procedure terrifies me.	**.799**		
(12) I dread walking in graveyards.	**.692**		
(13) I am preoccupied with thinking about what will happen after death.	**.509**	**.517**	
(14) I am afraid of sleeping and not waking up again.			**.696**
(15) The pain accompanying death terrifies me.		**.749**	
(16) I get upset by witnessing a funeral.	**.854**		
(17) The sight of a dying person frightens me.	**.769**		
(18) Talking about death upsets me.	**.766**		
(19) I am afraid of getting cancer.		**.888**	
(20) I fear death.			**.652**
Eigen value	9.94	2.39	1.24
% of variance	49.71	11.95	6.20
% of total variance	67.88		

Factor 1 (items: (2), (8), (11), (12), (13), (16), (17), and (18)): fear of dead people and tombs.

Factor 2 (items: (4), (5), (6), (7), (10), (13), (15), and (19)): fear of lethal disease and postmortem events.

Factor 3 (items: (1), (3), (14), and (20)): death fear.

## References

[1] Templer D. I. (1970). The contruction and validation of a Death Anxiety Scale. *The Journal of General Psychology*.

[2] Dadfar M., Lester D. (2015). Death concern and death obsession in iranian nurses. *Psychological Reports*.

[3] Sarıçiçek Aydoğan A., Gülseren Ş., Öztürk Sarıkaya Ö., Özen Ç. (2015). Reliability and validity of the Turkish version of Abdel-Khalek's Death Anxiety Scale among college students. *Archive of Neuropsychiatry*.

[4] Lester D., Neimeyer R. A. (1994). The Collett-Lester fear of death scale. *Death Anxiety Handbook: Research, Instrumentation and Application*.

[5] Lester D. (2004). The factorial structure of the revised collett-lester fear of death scale. *Death Studies*.

[6] Lester D., Abdel-Khalek A. M. (2003). The collett-lester fear of death scale: a correction. *Death Studies*.

[7] Dadfar M., Lester D., Atef Vahid M. K., Asgharnejad Farid A. A., Birashk B. (2015). *Death Distress in Nurses: Psychoeducational Interventions*.

[8] Bahrami F., Dadfar M., Lester D., Abdel-Khalek A. M. (2014). Death distress in Iranian older adult. *Advances in Environmental Biology*.

[9] Dadfar M., Bahrami F., Sheybani Noghabi F., Askari M. (2016). Relationship between religious spiritual well-being and death anxiety in Iranian elders. *International Journal of Medical Research & Health Sciences (IJMRHS)*.

[10] Gire J. T., Lonner W. J., Dinnel D. L., Hayes S. A., Sattler D. N. (2002). How death imitates life: cultural influences on conceptions of death and dying. *Online Readings in Psychology and Culture (Unit 14, Chapter 2)*.

[11] Lester D., Templer D. I., Abdel-Khalek A. (2006-2007). A cross-cultural comparison of death anxiety: a brief note. *Omega*.

[12] Maxfield M., Kluck B., Greenberg J. (2013). Age-related differences in responses to thoughts of one’s own death: mortality salience and judgments of moral transgressions. *Psychology Aging*.

[13] Dadfar M., Lester D., Asgharnejad Farid A. A., Atef Vahid M. K., Birashk B. (2014). Death depression in Iranian nurses. *Advances in Environmental Biology*.

[14] Dadfar M., Lester D., Asgharnizad Farid A. A., Atef Vahid M. K., Birashk B. (2014). Reasons for fearing death in Iranian nurses. *Global Journal on Advances in Pure and Applied Sciences*.

[15] Dadfar M., Asgharnejad Farid A. A., Lester D., Atef Vahid M. K., Birashk B. (2016). Effectiveness of death education program by methods of didactic, experimental, and 8A model on the reduction of death distress among nurses. *International Journal of Medical Research & Health Sciences*.

[16] Missler M., Stroebe M., Geurtsen L., Mastenbroek M., Chmoun S., Van Der Houwen K. (2011). Exploring death anxiety among elderly people: a literature review and empirical investigation. *Omega*.

[17] Momtaz Y. A., Haron Sh. A., Ibrahim R., Hamid T. A. (2015). Spousal death anxiety in old age: gender perspective. *Omega*.

[18] Dadfar M., Lester D. (2014). *Death Education Program: A Practical Guide for Healthcare Professionals*.

[19] Dadfar M., Lester D. (2014). Fear of death in Iranian nurses. *The Neuroscience Journal of Shefaye Khatam*.

[20] Anvar M., Javadpour A., Zadeh S. M. (2012). Assessing death anxiety and its correlates among severe medically ill in- patients. *Shiraz E Medical Journal*.

[21] Ghorbanalipoor M., Borjali A., Sohrabi F., Falsafinejad M. R. (2010). Effect of death anxiety and age on health promoting behaviors. *The Journal of Urmia University of Medical Sciences*.

[22] Abdel-Khalek A. M., Lester D. (2009). Death anxiety as related to somatic symptoms in two cultures. *Psychological Reports*.

[23] Ayyad F. (2013). Death distress among two samples of lower and higher stress in health care professionals. *Psychological Reports*.

[24] Daradkeh F., Moselhy H. F. (2011). Death anxiety (Thanatophobia) among drug dependents in an Arabic psychiatric hospital. *The American Journal of Drug and Alcohol Abuse*.

[25] Abdel-Khalek A. M. (2005). Death anxiety in clinical and non-clinical groups. *Death Studies*.

[26] Abdel-Khalek A. M., Al-Kandari Y. (2007). Death anxiety in Kuwaiti middle-aged personnel. *Omega*.

[27] Abdel-Khalek A. M. (2004). A general factor of death distress in seven clinical and non-clinical groups. *Death Studies*.

[28] Abdel-Khalek A. M. (2004). The Arabic Scale of Death Anxiety (ASDA): its development, validation, and results in three Arab countries. *Death Studies*.

[29] Abdel-Khalek A., Lester D., Maltby J., Tomás-Sábado J. (2009). The Arabic scale of death anxiety: some results from East and West. *Omega*.

[30] Neimeyer R. A., Fortner B. V., Maddox G. (1995). Death anxiety in the elderly. *Encyclopaedia of Aging*.

[31] Suhail K., Akram S. (2002). Correlates of death anxiety in Pakistan. *Death Studies*.

[32] Madnawat A. V. S., Kachhawa P. S. (2007). Age, gender, and living circumstances: discriminating older adults on death anxiety. *Death Studies*.

[33] Singh R. S. (2013). Death anxiety among aged Manipuris, India. *ZENITH International Journal of Multidisciplinary Research*.

[34] Thabet A. A., Tawahina A. A., Sarraj E. E., Vostanis P. (2013). Death anxiety, PTSD, trauma, grief, and mental health of Palestinian victims of war on Gaza. *Health Care Current Reviews*.

[35] Fortner B. V., Neimeyer R. A. (1999). Death anxiety in older adults: a quantitative review. *Death Studies*.

[36] Wu A. M. S., Tang C. S. K., Kwok T. C. Y. (2002). Death anxiety among Chinese elderly people in Hong Kong. *Journal of Aging and Health*.

[37] Chuin C. L., Choo Y. C. (2010). Age, gender, and religiosity as related to death anxiety. *Sunway Academic Journal*.

[38] Mimrot B. H. (2011). A comparative study of death anxiety of old persons. *Indian Streams Research Journal*.

[39] Rasmussen C. H., Johnson M. E. (1994). Spirituality and religiosity: relative relationships to death anxiety. *Omega; Journal of Death and Dying*.

[40] Schumaker J. F., Barraclough R. A., Vagg L. M. (1988). Death anxiety in Malaysian and Australian university students. *The Journal of Social Psychology*.

[41] DePaola S. J., Griffin M., Young J. R., Neimeyer R. A. (2003). Death anxiety and attitudes toward the elderly among older adults: the role of gender and ethnicity. *Death Studies*.

[42] Keller J. W., Sherry D., Piotrowski C. (1984). Perspectives on death: a developmental study. *The Journal of Psychology*.

[43] Rasmussen C. A., Brems C. (1996). The relationship of death anxiety with age and psychosocial maturity. *The Journal of Psychology*.

[44] Wagner K. D., Lorion R. P. (1984). Correlates of death anxiety in elderly persons. *Journal of Clinical Psychology*.

[45] Wink P., Scott J. (2005). Does religiousness buffer against the fear of death and dying in late adulthood? Findings from a longitudinal study. *Journals of Gerontology—Series B Psychological Sciences and Social Sciences*.

[46] Lester D., Abdel-Khalek A. M. (2008). Religiosity and death anxiety using non-Western scales. *Psychological Reports*.

[47] Wen Y.-H. (2010). Religiosity and death anxiety. *The Journal of Human Resource and Adult Learning*.

[48] Pyne D. http://www.sciencedirect.com/science/article/pii/1053535709001140.

